# Effect of Cattle on *Salmonella* Carriage, Diversity and Antimicrobial Resistance in Free-Ranging Wild Boar (*Sus scrofa*) in Northeastern Spain

**DOI:** 10.1371/journal.pone.0051614

**Published:** 2012-12-17

**Authors:** Nora Navarro-Gonzalez, Gregorio Mentaberre, Concepción M. Porrero, Emmanuel Serrano, Ana Mateos, José M. López-Martín, Santiago Lavín, Lucas Domínguez

**Affiliations:** 1 Servei d’ Ecopatologia de Fauna Salvatge (SEFaS), Departament de Medicina i Cirurgia Animals, Universitat Autònoma de Barcelona (UAB), Bellaterra, Barcelona, Spain; 2 Centro de Vigilancia Sanitaria Veterinaria (VISAVET), Faculty of Veterinary Medicine, Universidad Complutense (UCM), Madrid, Spain; 3 Estadística i Investigació Operativa, Departament de Matemàtica, Universitat de Lleida (UdL), Lleida, Spain; 4 Àrea d’Activitats Cinegètiques, Direcció General del Medi Natural i Biodiversitat, Generalitat de Catalunya, Barcelona, Spain; University College Dublin, Ireland

## Abstract

*Salmonella* is distributed worldwide and is a pathogen of economic and public health importance. As a multi-host pathogen with a long environmental persistence, it is a suitable model for the study of wildlife-livestock interactions. In this work, we aim to explore the spill-over of *Salmonella* between free-ranging wild boar and livestock in a protected natural area in NE Spain and the presence of antimicrobial resistance. *Salmonella* prevalence, serotypes and diversity were compared between wild boars, sympatric cattle and wild boars from cattle-free areas. The effect of age, sex, cattle presence and cattle herd size on *Salmonella* probability of infection in wild boars was explored by means of Generalized Linear Models and a model selection based on the Akaike’s Information Criterion. Prevalence was higher in wild boars co-habiting with cattle (35.67%, CI 95% 28.19–43.70) than in wild boar from cattle-free areas (17.54%, CI 95% 8.74–29.91). Probability of a wild boar being a *Salmonella* carrier increased with cattle herd size but decreased with the host age. Serotypes Meleagridis, Anatum and Othmarschen were isolated concurrently from cattle and sympatric wild boars. Apart from serotypes shared with cattle, wild boars appear to have their own serotypes, which are also found in wild boars from cattle-free areas (Enteritidis, Mikawasima, 4:b:- and 35:r:z35). Serotype richness (diversity) was higher in wild boars co-habiting with cattle, but evenness was not altered by the introduction of serotypes from cattle. The finding of a *S*. Mbandaka strain resistant to sulfamethoxazole, streptomycin and chloramphenicol and a *S*. Enteritidis strain resistant to ciprofloxacin and nalidixic acid in wild boars is cause for public health concern.

## Introduction

Interactions in the wildlife-livestock interface are currently increasing in the EU since animal husbandry is moving from more intensive to more extensive farming systems [1]. This fact often enhances disease transmission between wildlife and livestock and may be of particular concern in relation to wild ungulates, which frequently share habitat resources with domestic livestock [2]. The wild boar is especially considered a carrier and reservoir of several zoonotic pathogens [3], [4].

Among the pathogens shared between wildlife and domestic animals, little is known about *Salmonella* spp. (one of the most common genera of zoonotic bacteria of worldwide economic and health importance [5]) and the role of the wildlife-livestock interface in its transmission. This microorganism is considered a true multi-host pathogen with a long environmental persistence [6]. These characteristics (broad host range of some serotypes and long environmental persistence) make it a suitable model for studying interactions between wildlife and livestock in natural environments.

Most of the studies on *Salmonella* in wildlife focus on vectors such as insects, rodents and birds in the farm environment (some examples are [7], [8], [9]), and to our knowledge only one study relates the *Salmonella* prevalence and serotypes in wild large mammals to those found in co-habiting livestock: Glawischnig and colleagues [10] report an outbreak of salmonellosis (caused by serotype Dublin) in chamois (*Rupicapra rupicapra*) which had its origin in sick cattle grazing in the same pasture.

From a public health perspective, wildlife can play an important role in the complex *Salmonella*-wildlife-human cycle [11] since wildlife has been shown to be a common reservoir of this pathogen, in addition, *Salmonella* can be isolated at virtually every step of the game meat chain [12] and healthy animals can shed *Salmonella* over long periods of time. *Salmonella* is also of public health concern because many strains are resistant to a number of antimicrobial agents: data show that 44% of the *Salmonella* samples isolated from animal slaughter and veterinary diagnostic sources were resistant to at least one antimicrobial agent [13].

In wildlife, investigations of antimicrobial resistance are highly variable in their results, mainly depending on the host species, the bacterial species and the geographic location, but it is assumed that livestock and humans may be sources of antimicrobial resistance in wildlife (some examples, though not of *Salmonella*, are [14], [15], [16]).

It has been stated that an ecological approach may help to understand pathogen dynamics and host-pathogen interactions, but few studies have actually applied tools from population ecology to environmental microbiology and veterinary research (some examples on *Salmonella* are [17], [18] or [19]). This may be essential from a public health perspective, e.g. [20] found that asymptomatic *Salmonella* Typhimurium DT104 isolates showed a greater phenotypic and genotypic diversity within pig herds than disease-associated ones. Especially in the case of research in wildlife diseases, this multidisciplinary approach must be adopted [21], [22].

In this work, we aim to assess the effect of livestock presence on the prevalence and the components of diversity (richness and evenness) of *Salmonella* in the free-ranging wild boar population in the Ports de Tortosa i Beseit National Game Reserve, northeast Spain. In this game reserve, cattle occupy specific areas during the greater part of the year. Also, we assessed antimicrobial resistance in both wild boar and cattle in this natural area, where human activities are minimal.

## Materials and Methods

### Study Area

**Figure 1:**
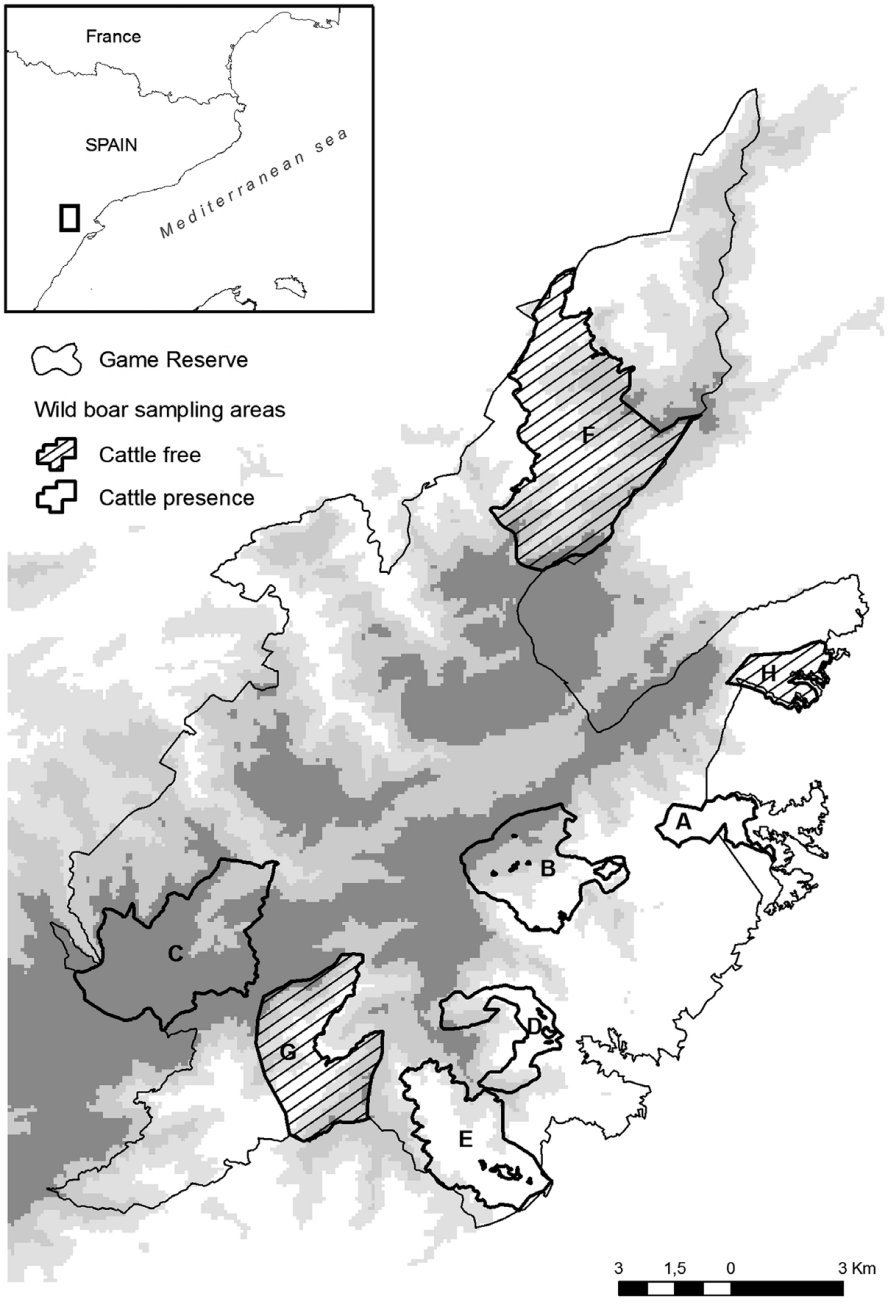
Sampling areas with cattle presence and absence.

The study area is located within the National Game Reserve Els Ports de Tortosa i Beseit in northeastern Spain, which is also part of the Natural Park of the same name. It is a calcareous mountain region with high orographic complexity that results in a rugged and abrupt terrain with numerous ravines and steep slopes. About 28% of the surface is above 1000 m.o.s.l., with the highest peak being Mont Caro (1442 m). The predominant habitat is pine grove (39%) followed by oak grove (15%), and, due to the dry Mediterranean climate, rivers account for only 0.2%. The most abundant wild ungulates are the Iberian ibex (*Capra pyrenaica*) and the wild boar (*Sus scrofa*), which are exploited for hunting purposes. Wildlife and cattle share pastures in some valleys of the study area, therefore, we chose three hunting areas (HA, hereafter) that are free of cattle presence and five HA are grazed by cattle either year-round or during the hunting season (see Figure 1: areas have been called A to H). Areas were categorised as grazed or cattle-free, and the animals were grouped according to this category for some analyses. Despite all HA belong to the same ecosystem and the ubiquitous nature of *Salmonella* and its high survival rate in soil [23]; we took into account that differences between areas in the landscape composition could have confounding effects on the results. Therefore, we checked for differences among HA by Geographic Information System (GIS) analysis. In brief, we have used CORINE land cover data CLC2006 [24] with a working scale of 1∶100.000, a minimum mapping unit 25 hectares and a minimum width of linear elements 100 metres. Later we estimated the surface of the main landscape classes using the ArcGIS 10 (Esri® ArcMap 10) by area and a MANOVA analysis for comparing landscape features (e.g., mean slope, mean altitude, percentage of sclerophyllous vegetation, percentage of mixed forest, percentage of coniferous forest and percentage of transitional woodland-shrub, all of them as response variables) between two categories (grazed vs ungrazed, as explanatory factor). No difference in terms of land use and landscape composition was observed among our sampling areas (Pillai statistic = 0.93, df = 1 p = 0.47), hence we assume that landscape characteristics will have minimum effect on the observed patterns of *Salmonella* occurrence.

### Animal Sampling

#### Wild boar

Altogether, 214 individual faecal samples were obtained from hunter-harvested wild boars during the regular hunting season (October to January) from 2007–2008 to 2010–2011. Fifty-seven samples were obtained from the cattle-free HA and 157 from the grazed ones. The difference in sample size is due to a different hunting effort (mean hunting days/year in HA with cattle is 23, while it is 8 in cattle-free HA, F = 117.48 df = 1, p<0.001, ANOVA) owing to easier access and orography in grazed areas, which results in a higher amount of wild boars hunted in those areas each year (mean number of wild boars hunted/year in cattle free HA is 50, while it is 110 in HA with cattle, F = 7.41, df = 1, p = 0.01, ANOVA).

On the other hand, to collect further information on the animals, sex was visually determined by direct observation of genitalia and age was estimated based on tooth eruption pattern and replacement as well as dental attrition [25]. So as to minimize error in age determination, four age classes were used: piglets (up to 5 months), juveniles (6 to 12 months), yearlings (between 13 and 24 months) and adults (over 24 months). Finally, Juveniles and Piglets were grouped together due to the small sample size of these age classes. Faeces were collected directly from the rectum and stored in sterile containers. They were refrigerated and sent to the laboratory within the following 24 hours.

#### Cattle

Seventy-three cattle samples were collected from 2008 to 2010. Previously, information was obtained from the Reserve’s managers on herd location (five herds, size 30, 50, 60, 70 and 170 individuals). The farming conditions in the National Game Reserve are free-ranging with supplemental feeding in the dry season (summer) and herds are small, which sometimes made it difficult to locate the animals (e.g., 60 animals in a 1823 ha area). The herd in area A (see Fig. 1) belongs to a bullfighting breed while the rest (B – E) are herds aimed at meat production. Cattle sampling was preferably performed on days that wild boars were also sampled. When cattle were located, animals were counted to assess aggregation and observed until defecation. Then, faeces were stored in a sterile container and refrigerated and sent to the laboratory within the following 24 hours.

#### Ethics statement

No permit or approval was needed for this work, since it does not imply extraordinary activities in the National Game Reserve. Faecal samples from the animals were collected specifically for this study. All animals were legally hunted and sampled with the permission of the National Game Reserve. Wild boars are hunted by groups of local hunters by the traditional method of this region (drive hunting) as allowed by the National Game Reserve. Hunters allowed the sampling of the harvested animals and the use of these samples for scientific purposes. Cattle were also sampled on public land and since samples were environmental there was no need for animal management.

### Microbiological Analyses

Cultures of *Salmonella* were performed according to ISO 6579∶2002 Annex D [26],which is the method recommended by the European Union Reference Laboratory for *Salmonella* in faecal and environmental samples. For all samples a 1/10 dilution in buffered peptone water (BPW) was made, then incubated at 37°C±1°C for 18 h±2 h. Next, Modified semi-solid Rappaport-Vassiliadis (MSRV) (Difco) agar plates were inoculated with three drops (a total volume of 0.1 ml) of BPW culture. Plates were incubated at 41.5°C±1°C for 24 h±3 h and if negative, they were incubated for an additional 24 h±3 h. Suspected growth of *Salmonella* was confirmed by plating on both Xylose Lysine Desoxycholate agar (XLD) (bioMérieux) and on chrom ID™ *Salmonella* agar (SM ID2) (bioMérieux). The plates were incubated for 24 h±3 h at 37°C±1°C.

Colonies of presumptive *Salmonella* were subcultured on Columbia 5% sheep blood agar (bioMérieux) and incubated for 24 h±3 h at 37°C±1°C. Identity of isolates as *Salmonella* spp. was confirmed by a commercially available biochemical method Enterotube ™ II (BD BBL ™). Serological typing of one isolate per sample was performed based on the Kauffmann-White scheme [27].

Phage Typing was performed at the National Center of Microbiology, Institute of Health Carlos III (Madrid, Spain) by using Anderson’s scheme [28].

### Antimicrobial Resistance Testing

A set of isolates was selected in order to characterize the frequency of antimicrobial resistance within each host group (cattle, wild boars from cattle-free areas, wild boars from grazed areas). At least one isolate from each serotype within each host group was tested for antimicrobial resistance. Most serotypes were isolated only once within each host group. When serotypes were more frequent, additional isolates were tested only when they belonged to different places or times with respect to the isolate already tested.

Antimicrobial resistance was tested by the agar diffusion method to obtain the Inhibition Zone Diameter (IZD) against amoxicillin-clavulanate, cefoxitin, amikacin, apramicin, imipenem and aztreonam while the broth microdilution method was performed to determine the minimum inhibitory concentrations (MIC) of sulfamethoxazole, gentamicin, ampicillin, ciprofloxacin, cefotaxime, ceftazidime, tetracycline, streptomycin, trimethoprim, chloramphenicol, florfenicol, kanamycin and nalidixic acid. Cut-off/breakpoint values are shown in Table 1.

**Table 1:** Antimicrobial agents used and cut-off values.

Method	Antimicrobial agent	Disk content/concentration range	Cut-off value/Break-point	Reference
Disk diffusion	Amoxicillin-clavulanate	30 µg	14 mm	VAV 2005
	Cefoxitin	30 µg	15 mm	CLSI
	Amikacin	30 µg	15 mm	CLSI
	Apramicin	20 µg	20 mm	Rosco diagnostica
	Imipenem	10 µg	20 mm	CLSI
	Aztreonam	30 µg	18 mm	CLSI
Broth microdilution	Sulfamethoxazole	8–1024 µg/ml	256 µg/ml	EFSA
	Gentamicin	0.25–32 µg/ml	2 µg/ml	EFSA
	Ampicillin	0.5–32 µg/ml	8 µg/ml	EFSA
	Ciprofloxacin	0.008–8 µg/ml	0.064 µg/ml	EFSA
	Cefotaxime	0.06–4 µg/ml	0.5 µg/ml	EFSA
	Ceftazidime	0.25–16 µg/ml	2 µg/ml	EUCAST
	Tetracycline	1–64 µg/ml	8 µg/ml	EFSA
	Streptomycin	2–128 µg/ml	16 µg/ml	EFSA
	Trimethoprim	0.5–32 µg/ml	2 µg/ml	EFSA
	Chloramphenicol	2–64 µg/ml	16 µg/ml	EFSA
	Florfenicol	2–64 µg/ml	16 µg/ml	EUCAST
	Kanamycin	4–128 µg/ml	32 µg/ml	CLSI
	Nalidixic acid	4–64 µg/ml	16 µg/ml	EFSA

### Statistical Analysis

To assess the effect of cattle on *Salmonella* infection probability in wild boar, we fitted a set of independent generalized linear models (GLM) using a binomial distribution and the logit link function [29] in which the response variable was explained by the single and the additive effects of age, sex, cattle presence and cattle herd size, and their two-way interactions. Herd size was included as an explicative variable, since it has been previously shown to be related to *Salmonella* prevalence and shedding [13]. Cattle density was not considered because it is too low in our study area and shows little variation (range 0.02–0.42 adult cow/ha). Wild boar abundance, which would be a likely factor affecting *Salmonella* infection in wild boar, was not different between areas with and without cattle presence (F = 0.04, df = 1, p = 0.84, ANOVA) and was therefore not included in the models. Complete information allowing for statistical analyses was known for 204 animals. Animals sampled from regular hunting activity are assumed to be representative of the healthy population [30]).

For all statistical models, we performed a model selection procedure based on the information-theoretic approach and the Akaike’s Information Criterion corrected for small sample sizes (AICc) [31], [32]. In short, competing models are ranked in relation to the difference between their Akaike scores with the score of the best model (Δi), which has the lowest AICc. Models with Δi <2 units have substantial support for explaining the observed variability in the variable of interest. Subsequently, we estimated the Akaike weight (*w*_i,_), defined as the relative probability that a given model is the best model among those being compared. Once the best model was selected, the explained deviance (ED) was calculated as a measure of explained variability of each response variable [33]. Additional recommended readings for guidance are [34] and [35].

Diversity was compared between host populations by means of its components: richness and evenness. Richness is in this case the number of serotypes found in each host group, while evenness is the relative distribution of isolates among serotypes. Evenness was assessed by the probability of interspecific encounter (PIE), which is defined as the probability that two randomly sampled individuals from the assemblage represent different species [36] (i.e., *Salmonella* isolates are “individuals” and serotypes are “species”).

We faced the problem that richness strongly depends on sample size [36], therefore, it could not be directly compared. Richness was corrected for sample size with the use of bootstrapping. The statistical analyses were performed using R software version 2.15.1 [37], including prevalence estimates, which were estimated by package “epiR” 0.9–43 version [38], and EcoSim 7.72 [39].

## Results

### Wild Boar

The prevalence of *Salmonella* among wild boars from cattle-free areas (n = 57) was 17.54% (CI 95% 8.74–29.91) (see serotypes and antimicrobial resistance in Table 2). Their counterparts with contact with cattle showed prevalence two times higher (35.67% CI 95% 28.19–43.70, n = 157, see Table 3), with this difference being statistically significant (χ^2^ = 5.62, df = 1, p = 0.02). However, all animals from both groups were apparently healthy.

**Table 2:** Serotypes and antimicrobial resistance of *Salmonella* isolates from wild boar from cattle-free areas in a Natural Park in northeastern Spain.

Serotype	Number of isolates	Area	Number of antibiograms	Antimicrobial resistance
**4:b:-**	3	F,G	2	Susceptibility
**Enteritidis**	1	H	1	CIPR, NAL Susceptibility Susceptibility
	1	H	1	Susceptibility
**Ohio**	1	G	1	Susceptibility
**42:l,v:z**	1	H	1	Susceptibility
**Shangai**	1	F	1	Susceptibility
**Mikawasima**	1	H	1	Susceptibility
**35:r:z35**	1	F	1	Susceptibility

CIPR = Ciprofloxacin, NAL = Nalidixic acid.

**Table 3:** Serotypes and antimicrobial resistance of *Salmonella* isolates from hunted wild boars in areas with cattle presence in a Natural Park in northeastern Spain.

Serotype	Number of isolates	Area	Number of antibiograms	Antimicrobial resistance
**Meleagridis**	13	A	13	Susceptibility
**4:b:-**	8	A,B,D	4	Susceptibility
**Muenster**	6	A,B	3	Susceptibility
**42:b:e,n,x,z15**	4	D,C	2	Susceptibility
**Newport**	3	A	2	Susceptibility
**Anatum**	2	A	1	Susceptibility
**Othmarschen**	2	A	1	Susceptibility
**Carnac**	2	A, B	2	Susceptibility
**16:l,v:1,5,7**	2	A,D	1	Susceptibility
**Stoneferry**	1	A	1	Susceptibility
**Stanley**	1	A	1	Susceptibility
**Spartel**	1	A	1	Susceptibility
**Offa**	1	A	1	Susceptibility
**Mikawasima**	1	A	1	Susceptibility
**Mbandaka**	1	A	1	SMX, STR, CHL
**Kottbus**	1	A	1	Susceptibility
**Enteritidis**	1	A	1	Susceptibility
**58:K:-**	1	A	1	Susceptibility
**48:**	1	D	1	Susceptibility
**38:l,v:z54**	1	A	1	Susceptibility
**38:l,v:z53**	1	E	1	Susceptibility
**Tomegbe**	1	A	1	Susceptibility
**Paratyphi B**	1	A	1	Susceptibility

SMX = Sulfamethoxazole, CHL = Chloramphenicol, STR = Streptomycin.

### Cattle

The *Salmonella* prevalence among cattle was 21.92% (CI 95% 13.10–33.14, n = 73). See Table 4 for information about serotypes and antimicrobial susceptibility.

**Table 4:** Serotypes and antimicrobial resistance of *Salmonella* from cattle from a Natural Park in northeastern Spain.

Serotype	Number of isolates	Herd size	Number of antibiograms	Antimicrobial resistance
**Anatum**	10	170	3	Susceptibility
**Meleagridis**	4	170	4	Susceptibility
**Kedougou**	1	170	1	Susceptibility
**Othmarschen**	1	50	1	Susceptibility

### Antimicrobial Resistance

Two strains (2.98%) showed antimicrobial resistance; one *Salmonella* Enteritidis and one *Salmonella* Mbandaka strain (see Tables 2 and 3). The resistant *Salmonella* Enteritidis was carried by a wild boar from a cattle-free area (H), and showed resistance against ciprofloxacin and nalidixic acid. To the contrary, the *Salmonella* Mbandaka strain was carried by a wild boar from the cattle-grazed area A and was resistant to sulfamethoxazole, streptomycin and chloramphenicol.

### Inter-specific Overlap of *Salmonella* Serotypes

A wide variety of *Salmonella* serotypes are shown in Tables 2, 3, 4 and 5. Some serotypes have been found in both wild boar from cattle-free and cattle-grazed areas (Enteritidis, 4:b:-, Mikawasima and 35:r:z35). Serotypes 4:b:- and Enteritidis were the most frequent found in wild boars from cattle-free areas (30% and 20% of the total isolates, respectively), while they were present in a lower frequency in wild boars from cattle-grazed areas (1.79% and 14.29%, respectively). Cattle serotypes were only shared with wild boars from cattle-grazed areas (e.g., Meleagridis, the most frequent serotype in this group was the second most frequent in cattle, or Anatum and Othmarschen also appeared in sympatric wild boars). This overlap suggests some degree of spill-over between cattle and wild boar. The fact that these serotypes were simultaneously isolated from both host species in the same place (A) and its PFGE pattern (see [40]) indicate a direct association between *Salmonella* isolates from cattle and wild boar.

**Table 5:** Model selection for the probability of *Salmonella* carriage in wild boars.

Biological Models	*K*	AICc	Δi	*w*i
**Herd size + age class**	**4**	**234.02**	**0.00**	**0.51**
Herd size * age class	**6**	**234.30**	**0.28**	**0.45**
Herd size	2	240.17	6.15	0.02
Herd size + sex	3	241.64	7.63	0.01
Herd size * sex	4	243.70	9.68	0.00
Cattle presence + age class	4	249.40	15.38	0.00
Cattle presence * age class	6	250.09	16.07	0.00
Cattle presence	2	252.45	18.43	0.00
Age class	3	254.16	20.14	0.00
Cattle presence + sex	3	254.40	20.38	0.00
Sex + age class	4	256.20	22.18	0.00
Cattle presence * sex	4	256.21	22.19	0.00
Null	1	257.36	23.34	0.00
Sex * age class	6	258.22	24.20	0.00
Sex	2	259.18	25.16	0.00

*K* = number of parameters, AICc = Akaike’s Information Criterion corrected for small sample sizes, Δi = difference of AICc with respect to the best model, *w*i = Akaike weight. In bold, models with substantial support.

Phage-typing of *Salmonella* serotype Enteritidis strains revealed no association between these isolates: the strain from area A showed an unrecognizable lytic pattern, while the strains from the cattle-free area H were PT1 and 14C.

### *Salmonella* Serotype Richness and Evenness

In the smallest group, i.e. wild boars from cattle-free areas, serotype richness was 7 (See Table 2). This value had to be compared with serotype richness in cattle and in wild boars from cattle-grazed areas at a sample size equal to 57. Serotype richness was clearly lower in cattle: 4 serotypes was the maximal value (this means that the probability of being smaller than 7 is 100%) and also the most probable (610 out of 1000 repetitions, i.e. probability _(Richness = 4) = _0.61). The group of wild boars from cattle-grazed areas had 12 as a most probable value (p _(Richness = 12) = _0.22), and probability that its richness was greater than 7 was 0.99.

These results indicate higher *Salmonella* serotype richness in wild boars from cattle-grazed areas than their cattle-free counterparts: when sampling an equal number of wild boars from both areas we would have a greater number of different serotypes in wild boars from grazed areas. However, richness in cattle was lower than in both groups of wild boars.

The index for evenness was lower in cattle (PIE _Cattle_ = 0.57) than in both groups of wild boars (PIE_1_ = 0.91, PIE_2_ = 0.92), indicating that two *Salmonella* isolates taken randomly from wild boars will be different serotypes with a probability higher than 0.9, while two *Salmonella* isolates randomly detected in cattle will differ with a probability of 0.57. Hence a certain serotype seems to be predominant in cattle, but this is not the case in wild boars despite their co-habiting with cattle.

### *Salmonella* Infection Probability in Wild Boar

**Figure 2:**
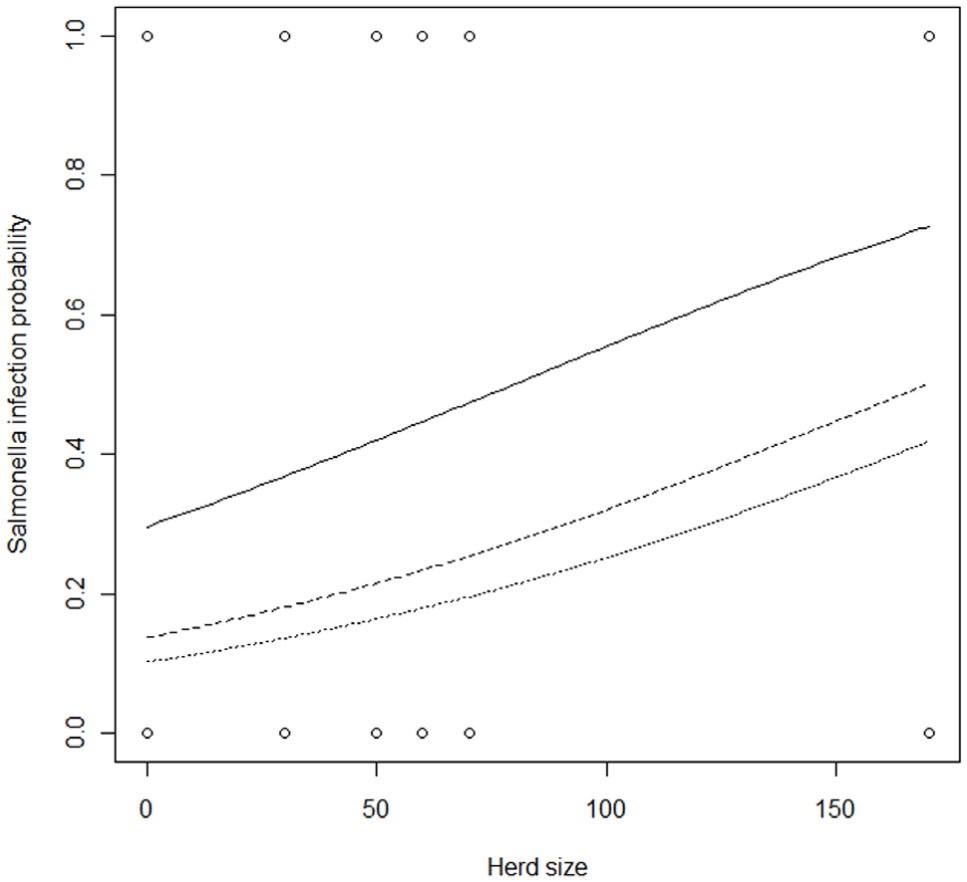
Relationship between *Salmonella* carriage probability in wild boar age classes and size of the cattle herd cohabiting in the area. Legend: solid line = piglets and juveniles (intercept = 0.3), dashed line = yearlings (intercept = 0.14), dotted line = adults (intercept = 0.1). Slope = 0.01.

The best model for explaining *Salmonella* probability of infection (see Table 5) in wild boars was the additive effects of cattle herd size and age class (β _Herd size_ = 0.011±0.002, n = 204, w_i = _0.51, explained deviance = 11.56%). As can be seen in Figure 2, *Salmonella* infection probability increases with herd size, but decreases with age, especially after the first year of life (piglets and juveniles). The pure effect of the variables was 7.53 and 2.85% for herd size and age class, respectively, and there was no shared deviance. The relative importance of the variables also supports our results (R_i__Herd size_ = 0.99, R_i Age class_ = 0.96, R_i Sex_ = 0.01, R_i Cattle presence_ = 0). The second model with substantial support (see Table 5, Δ_i_ <2, w_i = _0.51) was not selected due to the principle of Parsimony. The same trend was observed in cattle: the probability of a cow being a *Salmonella* carrier increased as herd size increased (z = 2.78, β_Herd size = _0.07, SE = 0.02, p<0.01, explained deviance = 23.68%).

## Discussion

The prevalence found in the wild boars with contact with cattle in our study (35.67%) is the highest described to date in this species in the literature. On the other hand our results confirm the presence of *Salmonella enterica* serotypes of medical importance in wildlife; e.g., Enteritidis, Newport and Mbandaka were among the 10 most frequent serotypes causing salmonellosis in humans in the EU, and specifically the *S*. Enteritidis phage-type 1 is among the most frequent [41]. Other serotypes found in the study area have also been related to human outbreaks (e.g., Anatum, Paratyphi B and Kottbus, among others); and salmonellosis caused by *Salmonella enterica* serotype Enteritidis has been diagnosed in sympatric Iberian ibex [42], with unknown consequences at the population level. The high prevalence of *Salmonella* in wild boars and the fact that wild boars shed this pathogen to a higher extent than wild ruminants [11] make it possible that wild boar plays an important role in the transmission and maintenance of *Salmonella* in the study area and likewise in similar multi-host systems.

The prevalence observed in the wild boars from cattle-free areas (17.54%) is similar to that found in wild boars from Portugal: 22.1%; serotypes Typhimurium and Rissen [43]. Lower prevalences have been described either from tonsils of wild boar in Switzerland (12%; serotypes Enteritidis, Veneziana and Stourbridge [4]) or from tissues of wild boars in northern Spain (7.5%; serotypes Worthington and 38:l,v:z35 [44]).

Since *Salmonella* prevalence in wild boar from cattle-grazed areas was higher than prevalence in cattle itself, *Salmonella* sources other than cattle, but linked to its presence, may exist. For example, other wild hosts (wild birds and rodents) attracted by free access to cattle feed or other domestic animals (dogs or cats) could play a role in the epidemiology of *Salmonella* in the study area. Wild boar may be exposed to numerous *Salmonella* sources more directly than cattle due to its omnivorous and opportunistic feeding habits [45] that include potential *Salmonella* carriers, such as mice and birds [9], and especially its rooting behaviour may favour the transmission through inhalation, a potential route of *Salmonella* infection in pigs [46].

Cattle seem to contribute to the *Salmonella* prevalence in wild boar by introducing their own serotypes to the environment, which can be regarded as an additive effect. We also found that *Salmonella* in cattle and in sympatric wild boars increased when the cattle herd size increased. Nevertheless, it should be considered that herd-related factors other than herd size, such as food, breed or health status may be related to the *Salmonella* prevalence. Also, although not having considered cattle density as an explanatory variable due to its little variation among herds in the study area (see Material and Methods section), the bullfighting herd in area A is not only the largest, but also that with the highest density. Another difference is that fighting bulls, although under extensive management conditions, have to be kept inside enclosures given that they are potentially dangerous. In this case, these enclosures are not an effective biosecurity barrier, as evidenced by the results. Indeed, shared serotypes and simultaneous isolation in cattle and wild boar occurred only in this hunting area A. Similarly, Skov and colleagues [47] found *Salmonella* in wildlife surrounding farms where and when *Salmonella* was also detected in livestock. This is important because even at a low prevalence, wildlife can be a source of *Salmonella* for livestock [48].

As shown, serotypes found in previous studies about *Salmonella* in wild boar differ widely from our isolates. The wide number of serotypes found in our study area and the fact that most serotypes were isolated only once during the study period or in one animal only may reflect 1) a high diversity of *Salmonella* sources within the Reserve; 2) a high heterogeneity in the exposure of the wild boars to these sources; 3) a low intra-specific transmission of these serotypes; or 4) the separation of wild boars and their *Salmonella* strains by natural barriers (e.g., steep terrain). Indeed, Methner and colleagues detected different epidemiological groups of *Salmonella* serotype Cholerasuis in wild boars from certain regions of Thuringia (Germany) that were separated by natural (mountains) or artificial barriers (arterial roads) [49].

This may explain why richness was higher in wild boars (both from cattle-grazed and cattle-free areas) than in cattle. Cattle are supposed to live in more homogenous conditions that determine exposure within herd (e.g., food and water), which is supported by the lower serotype richness found in cattle. The highest richness was found in wild boars from cattle-grazed areas, and this confirms an additive effect of cattle on the *Salmonella* of sympatric wild boars, as explained above with *Salmonella* prevalence. However, PIE was similar in wild boars from cattle-grazed and cattle-free areas, suggesting that wild boar may be a spill-over host: serotype evenness has not been altered by the acquisition of serotypes from cattle, which dominate in cattle but not in the *Salmonella* population from wild boars.

It should be noted that serotyping only one isolate per sample may have underestimated richness. However, for our aim (comparing richness between host groups) this methodology should suffice since the underestimation would be the same in each group. Additionally, the protocol, included enrichment to favour the detection of *Salmonella,* and enrichment media can have an effect on the strain/serotype detected as shown by other studies ([50], [51]). Thus, serotyping more than one isolate would not be more representative.

On the other hand, antimicrobial resistance is an anecdotal finding in wild boar. Interestingly, two very different patterns of resistance have been found and they are directly related to the origin of the animals. The *Salmonella* serotype Mbandaka strain resistant to chloramphenicol, sulfamethoxazole and streptomycin was carried by a wild boar from a cattle-grazed area. Streptomycin and sulfonamides are, along with others, antimicrobial agents for which veterinary-associated *Salmonella* isolates show the greatest percentage of resistance [13]. In fact, serotype Mbandaka is the most frequent (20%) serotype isolated from cattle in Spain [41]; therefore, although this serotype was not isolated from cattle in our study area, its origin is possibly linked to cattle or associated factors.

The resistance profile displayed by a *S*. Enteritidis strain found in a wild boar from a cattle-free area (ciprofloxacin and nalidixic acid) is of concern since ciprofloxacin belongs to the second generation of fluoroquinolones and is today the antimicrobial of choice for treatment of severe or invasive *Salmonella* infections in humans [52]. This resistance profile is not usually found in Spanish cattle [52] suggesting the existence of a different source of antimicrobial resistance in the National Game Reserve. This resistance profile is frequent in *S*. spp from fowl and pigs, both particularly in Spain [52], [53]; but to our knowledge this type of farming does not occur in the study area nor within a short distance; on the other hand, the highest levels of resistance among *S*. Enteritidis isolates from humans in 2010 were observed for nalidixic acid, 18.7%, and ciprofloxacin, 9.3% [52].

Caleja and colleagues [54] reported a high frequency of antimicrobial resistance among *S*. Typhimurium and *S*. Rissen isolates from wild boar in Portugal against ampicillin, amoxicillin-clavulanic acid, streptomycin, chloramphenicol, tetracycline, sulfonamides and trimethoprim-sulfamethoxazole. Behaviour of wild boar, especially their feeding habits, makes this species prone to pathogen exposure and may also be linked to antimicrobial resistance carriage. Therefore, it may be a suitable sentinel for *Salmonella* presence, prevalence and antimicrobial resistance in a natural environment.

### Conclusions

This study provides evidence of the association of *Salmonella* infection in free-ranging wild boar and herd size of sympatric cattle in extensive farming conditions. It also suggests that sources of *Salmonella* other than livestock exist in the National Game Reserve. The spill-over of *Salmonella* between cattle and wild boar, although increasing serotype richness in the latter, did not alter the evenness of the *Salmonella* population in wild boar and thus may be sporadic or a dead-end. The presence of antimicrobial resistance in isolates from wildlife of a protected area is a matter of concern.

## References

[1] Gortázar C, Ferroglio E, Höfle U, Frölich K, Vicente J (2007) Diseases shared between wildlife and livestock: A european perspective. Eur J Wildl Res 53: 241–256.

[2] Boehm M, White PCL, Chambers J, Smith L, Hutchings MR (2007) Wild deer as a source of infection for livestock and humans in the UK. Vet J 174: 260–276. 17258479 10.1016/j.tvjl.2006.11.003

[3] Meng XJ, Lindsay DS, Sriranganathan N (2009) Wild boars as sources for infectious diseases in livestock and humans. Philos Trans R Soc Lond B Biol Sci 364: 2697–2707. 19687039 10.1098/rstb.2009.0086 PMC2865094

[4] Wacheck S, Fredriksson-Ahomaa M, Konig M, Stolle A, Stephan R (2010) Wild boars as an important reservoir for foodborne pathogens. Foodborne Pathog Dis 7: 307–312. 19899962 10.1089/fpd.2009.0367

[5] Uzzau S, Brown DJ, Wallis T, Rubino S, Leori G, et al (2000) Host adapted serotypes of *Salmonella enterica* . Epidemiol Infect 125: 229–255. 11117946 10.1017/s0950268899004379 PMC2869595

[6] Murray CJ (1991) Salmonellae in the environment. Rev Sci Tech 10: 765–785. 1782428

[7] Davies RH, Breslin M (2003) Persistence of *Salmonella* Enteritidis phage type 4 in the environment and arthropod vectors on an empty free-range chicken farm. Environ Microbiol 5: 79–84. 12558590 10.1046/j.1462-2920.2003.00387.x

[8] Garber L, Smeltzer M, Fedorka-Cray P, Ladely S, Ferris K (2003) *Salmonella enterica* serotype Enteritidis in table egg layer house environments and in mice in US layer houses and associated risk factors. Avian Dis 47: 134–142. 12713168 10.1637/0005-2086(2003)047[0134:SESEIT]2.0.CO;2

[9] Liebana E, Garcia-Migura L, Clouting C, Clifton-Hadley FA, Breslin M, et al (2003) Molecular fingerprinting evidence of the contribution of wildlife vectors in the maintenance of *Salmonella* Enteritidis infection in layer farms. J Appl Microbiol 94: 1024–1029. 12752810 10.1046/j.1365-2672.2003.01924.x

[10] Glawischnig W, Khaschabi D, Schopf K, Schonbauer M (2000) An outbreak of *Salmonella* Dublin in chamois (*Rupicapra rupicapra*). Wien Tierarztl Monatsschr 87: 21–25.

[11] Hilbert F, Smulders FJM, Chopra-Dewasthaly R, Paulsen P (2012) *Salmonella* in the wildlife-human interface. Food Res Int 45: 603–608.

[12] Paulsen P, Smulders FJM, Hilbert F (2012) *Salmonella* in meat from hunted game: A central European perspective. Food Res Int 45: 609–616.

[13] Foley SL, Lynne AM (2008) Food animal-associated *Salmonella* challenges: Pathogenicity and antimicrobial resistance. J Anim Sci 86: E173–E187. 17878285 10.2527/jas.2007-0447

[14] Dolejska M, Cizek A, Literak I (2007) High prevalence of antimicrobial-resistant genes and integrons in *Escherichia coli* isolates from black-headed gulls in the Czech Republic. J Appl Microbiol 103: 11–19. 17584448 10.1111/j.1365-2672.2006.03241.x

[15] Literak I, Dolejska M, Radimersky T, Klimes J, Friedman M, et al (2010) Antimicrobial-resistant faecal *Escherichia coli* in wild mammals in central Europe: Multiresistant *Escherichia coli* producing extended-spectrum beta-lactamases in wild boars. J Appl Microbiol 108: 1702–1711. 19849769 10.1111/j.1365-2672.2009.04572.x

[16] Skurnik D, Ruimy R, Andremont A, Amorin C, Rouquet P, et al (2006) Effect of human vicinity on antimicrobial resistance and integrons in animal faecal *Escherichia coli* . J Antimicrob Chemother 57: 1215–1219. 16581916 10.1093/jac/dkl122

[17] Lankau EW, Bedon LC, Mackie RI (2012) *Salmonella* strains isolated from Galapagos iguanas show spatial structuring of serovar and genomic diversity. Plos One 7: e37302. 22615968 10.1371/journal.pone.0037302 PMC3353930

[18] Patton TG, Scupham AJ, Bearson SMD, Carlson SA (2009) Characterization of fecal microbiota from a *Salmonella* endemic cattle herd as determined by oligonucleotide fingerprinting of rDNA genes. Vet Microbiol 136: 285–292. 19091494 10.1016/j.vetmic.2008.10.032

[19] Santos FBO, Sheldon BW, Santos AA Jr, Ferket PR, Lee MD, et al (2007) Determination of ileum microbial diversity of broilers fed triticale- or corn-based diets and colonized by *Salmonella* . J Appl Poult Res 16: 563–573.

[20] Perron GG, Quessy S, Letellier A, Bell G (2007) Genotypic diversity and antimicrobial resistance in asymptomatic *Salmonella enterica* serotype Typhimurium DT104. Infect Genet Evol 7: 223–228. 17049315 10.1016/j.meegid.2006.09.003

[21] Daszak P, Cunningham AA, Hyatt AD (2000) Wildlife ecology - emerging infectious diseases of wildlife - threats to biodiversity and human health. Science 287: 443–449. 10642539 10.1126/science.287.5452.443

[22] Hudson PJ, Rizzoli A, Grenfell BT, Heesterbeek J, Dobson AP (2002) Ecology of wildlife diseases. Oxford: Oxford University Press.

[23] Winfield MD, Groisman EA (2003) Role of nonhost environments in the lifestyles of *Salmonella* and *Escherichia coli* . Appl Environ Microbiol 69: 3687–3694. 12839733 10.1128/AEM.69.7.3687-3694.2003 PMC165204

[24] European Environment Agency (2007) CLC2006 technical guidelines. EEA Technical report No. 17/2007.

[25] Boitani l, Mattei L (1992) Aging wild boar (S*us scrofa*) by tooth eruption. In: Spitz F, Janeau G, Gonzalez G, Aulagnier S, editors. Ongulés/Ungulates. Paris-Toulouse: S.F.E.P.M.-I.R.G. M: 419–421.

[26] International Organization for Standardization (2007) Detection of *Salmonella* spp. in animal faeces and in environmental samples from the primary production stage. 6579: 2002.

[27] Grimont PAD, Weill F, editors (2007) Antigenic formulas of the *Salmonella* serovars. Paris: WHO Collaborating Centre for Reference and Research on Salmonella, Insitut Pasteur.

[28] Anderson ES, Ward LR, Saxe MJ, de Sa JD (1977) Bacteriophage-typing designations of *Salmonella* Typhimurium. J Hyg 78: 297–300. 321679 10.1017/s0022172400056187 PMC2129838

[29] Zuur AF, Ieno EN, Walker NJ, Saveliev AA, Smith GM (2009) Mixed effects models and extension in ecology with R. New York: Springer. 1–530 p.

[30] Thulke H, Eisinger D, Freuling C, Froehlich A, Globig A, et al (2009) Situation-based surveillance: Adapting investigations to actual epidemic situations. J Wildl Dis 45: 1089–1103. 19901383 10.7589/0090-3558-45.4.1089

[31] Burnham KP, Anderson DR (2002) Model selection and multimodel inference: A practical information-theoretic approach. New York: Springer-Verlag. 1–488 p.

[32] Johnson DH (2002) The role of hypothesis testing in wildlife science. J Wildl Manage 66: 272–276.

[33] Zuur AF, Ieno EN, Smith GM (2007) Analysing ecological data. New York, USA: Springer. 1–672 p.

[34] Anderson DR, Burnham KP, Thomson WL (2000) Null hypothesis testing: Problems, prevalence, and an alternative. J Wildl Manage 64: 912–923.

[35] Anderson DR, Burnham KP, White GA (2001) Kullback -Leibler information in resolving natural resource conflicts when definitive data exist. Wildlife Soc B 29: 1260–1270.

[36] Hurlbert SH (1971) Nonconcept of species diversity - critique and alternative parameters. Ecology 52: 577–586. 28973811 10.2307/1934145

[37] R Development Core Team 2.14.0 (2011) A language and environment for statistical computing. R foundation for statistical computing, Vienna, Austria. http://www.R-project.org. Accessed 3 October 2011.

[38] Stevenson M, Nunes T, Sanchez J, Thornton R, Reiczigel J, et al.. (2012) epiR: An R package for the analysis of epidemiological data. R package version 0.9–43.

[39] Gotelli NJ, Entsminger GL. EcoSim 7.72. acquired intelligence, inc. http://www.uvm.edu/~ngotelli/EcoSim/EcoSim.html. Accessed 12 April 2012.

[40] Mentaberre G, Porrero MC, Navarro-Gonzalez N, Serrano E, Domínguez L, et al. Cattle drive *Salmonella* infection in the wildlife-livestock interface. Zoonoses Public Hlth, In press. 10.1111/zph.12028 23253262

[41] EFSA (2012) The European union summary report on trends and sources of zoonoses, zoonotic agents and food-borne outbreaks. EFSA Journal 10: 2597. 10.2903/j.efsa.2018.5500 PMC7009540 32625785

[42] Navarro-Gonzalez N, Velarde R, Porrero MC, Mentaberre G, Serrano E, et al. Lack of evidence of spill-over of *Salmonella enterica* between cattle and sympatric Iberian ibex (*Capra pyrenaica*) from a protected area in Catalonia, NE spain. Transbound Emerg Dis, In press. 10.1111/tbed.12037 23217161

[43] Vieira-Pinto M, Morais L, Caleja C, Themudo P, Torres C, et al (2011) *Salmonella* sp in game (*Sus scrofa* and *Oryctolagus cuniculus*). Foodborne Pathog Dis 8: 739–740. 21254910 10.1089/fpd.2010.0742

[44] Millan J, Aduriz G, Moreno B, Juste RA, Barral M (2004) *Salmonella* isolates from wild birds and mammals in the Basque country (Spain). Rev Sci Tech 23: 905–911. 15861885 10.20506/rst.23.3.1529

[45] Schley L, Roper TJ (2003) Diet of wild boar *Sus scrofa* in Western Europe, with particular reference to consumption of agricultural crops. Mamm Rev 33: 43–56.

[46] Fedorka-Cray P, Kelley L, Stabel T, Gray J, Laufer J (1995) Alternate routes of invasion may affect pathogenesis of *Salmonella* Typhimurium in swine. Infect Immun 63: 2658–2664. 7790082 10.1128/iai.63.7.2658-2664.1995 PMC173356

[47] Skov MN, Madsen JJ, Rahbek C, Lodal J, Jespersen JB, et al (2008) Transmission of *Salmonella* between wildlife and meat-production animals in Denmark. J Appl Microbiol 105: 1558–1568. 19146492 10.1111/j.1365-2672.2008.03914.x

[48] Carlson JC, Franklin AB, Hyatt DR, Pettit SE, Linz GM (2011) The role of starlings in the spread of *Salmonella* within concentrated animal feeding operations. J Appl Ecol 48: 479–486. 10.1016/j.vetpar.2011.03.050 21536385

[49] Methner U, Heller M, Bocklisch H (2010) *Salmonella enterica* subspecies *enterica* serovar Choleraesuis in a wild boar population in Germany. Eur J Wildl Res 56: 493–502.

[50] Singer RS, Mayer AE, Hanson TE, Isaacson RE (2009) Do microbial interactions and cultivation media decrease the accuracy of *Salmonella* surveillance systems and outbreak investigations? J Food Prot 72: 707–713. 19435216 10.4315/0362-028x-72.4.707

[51] Gorski L (2012) Selective enrichment media bias the types of *Salmonella enterica* strains isolated from mixed strain cultures and complex enrichment broths. PloS One 7: e34722. 22496847 10.1371/journal.pone.0034722 PMC3319605

[52] EFSA (2010) The community summary report on antimicrobial resistance in zoonotic and indicator bacteria from animals and food in the European union in 2008. EFSA Journal 8: 1658.

[53] VAV (2005) Red de vigilancia veterinaria de resistencias a antibióticos. Number 12. http://www.vigilanciasanitaria.es/vav/. Accessed 2012 July 18.

[54] Caleja C, de Toro M, Goncalves A, Themudo P, Vieira-Pinto M, et al (2011) Antimicrobial resistance and class I integrons in *Salmonella enterica* isolates from wild boars and bisaro pigs. Int Microbiol 14: 19–24. 22015698 10.2436/20.1501.01.131

